# Sequence Features of Drosha and Dicer Cleavage Sites Affect the Complexity of IsomiRs

**DOI:** 10.3390/ijms16048110

**Published:** 2015-04-10

**Authors:** Julia Starega-Roslan, Tomasz M. Witkos, Paulina Galka-Marciniak, Wlodzimierz J. Krzyzosiak

**Affiliations:** Department of Molecular Biomedicine, Institute of Bioorganic Chemistry, Polish Academy of Sciences, Noskowskiego 12/14 Str., 61-704 Poznan, Poland; E-Mails: staregaj@ibch.poznan.pl (J.S.-R.); tom.witkos@gmail.com (T.M.W.); pgalka@ibch.poznan.pl (P.G.-M.)

**Keywords:** miRNA precursor sequence, miRNA precursor structure, heterogeneity, homogeneity, Dicer, Drosha

## Abstract

The deep-sequencing of small RNAs has revealed that different numbers and proportions of miRNA variants called isomiRs are formed from single miRNA genes and that this effect is attributable mainly to imprecise cleavage by Drosha and Dicer. Factors that influence the degree of cleavage precision of Drosha and Dicer are under investigation, and their identification may improve our understanding of the mechanisms by which cells modulate the regulatory potential of miRNAs. In this study, we focused on the sequences and structural determinants of Drosha and Dicer cleavage sites, which may explain the generation of homogeneous miRNAs (in which a single isomiR strongly predominates) as well as the generation of heterogeneous miRNAs. Using deep-sequencing data for small RNAs, we demonstrate that the generation of homogeneous miRNAs requires more sequence constraints at the cleavage sites than the formation of heterogeneous miRNAs. Additionally, our results indicate that specific Drosha cleavage sites have more sequence determinants in miRNA precursors than specific cleavage sites for Dicer and that secondary structural motifs in the miRNA precursors influence the precision of Dicer cleavage. Together, we present the sequence and structural features of Drosha and Dicer cleavage sites that influence the heterogeneity of the released miRNAs.

## 1. Introduction

Currently, it is well proven that each miRNA gene gives rise to a population of heterogeneous products called isomiRs, which have variable lengths and end-sequences, rather than to a single mature miRNA [1,2,3]. The existence of isomiRs, which considerably increase the regulatory potential of miRNAs, has prompted researchers to identify factors that influence the generation of heterogeneous miRNAs. The results of biochemical analyses sand deep-sequencing data indicate that isomiRs arise primarily from imprecise cleavage by Drosha and Dicer [1,4,5,6], the nucleases generating mature miRNA ends. Apart from imprecise cleavage by Drosha and Dicer, several secondary processes may alter the cleavage outcome. These downstream processes include non-templated nucleotide addition in a small fraction of pre-miRNAs or miRNAs [7,8,9] and the limited degradation of miRNAs by cellular exonucleases [10,11,12]. In addition, some preference for Ago2 loading, which depends on the nature of the miRNA 5'-end nucleotide [13,14], fine-tunes the final miRNA heterogeneity.

Recent experimental studies have investigated the structural features of pri-miRNAs and pre-miRNAs that influence the generation of isomiRs [15,16]. In particular, the distance from structural motifs to a cleavage site within pri-miRNA and pre-miRNA was shown to affect the precision of Drosha and Dicer cleavage, respectively. Lately, analyses of sequences of the wide range of human pri-miRNAs allowed the identification of sequence motifs, localized either in the pri-miRNA flanking sequences or in a terminal loop, that discriminate between true pri-miRNAs and pri-miRNA-like structures [17]. Two other bioinformatics studies analyzing deep-sequencing data on *C. elegans* and mouse miRNAs have revealed nucleotide sequence preferences at the sites of Drosha and Dicer cleavage; *i.e.*, a preference for uridine (U) at the first position of a miRNA and a bias against guanosine (G) 3' to the Drosha and Dicer cleavage sites [5,18]. However, the question of whether the nucleotide sequence preferences at the sites of Drosha and Dicer cleavage are involved in generating either homogeneous or heterogeneous miRNAs has not yet been addressed.

Our study is the first to address the issue of the nucleotide sequence contribution at Drosha and Dicer cleavage sites to the formation of the homogeneous and heterogeneous miRNAs. We analyzed the nucleotide composition at Drosha and Dicer cleavage sites with the use of publicly available small RNA deep-sequencing data. The results of our analyses expand existing knowledge regarding sequence biases observed at miRNA ends. This observation is further analyzed in detail in the context of the homogeneity and heterogeneity of miRNA generated by Drosha and Dicer. We propose that nucleotide sequences at and around Drosha and Dicer cleavage sites may play an important role in determining the level of miRNA heterogeneity. Our results shed new light on the issue of the formation of the isomiRs, the abundance of which reflects the sequence preference of Drosha and Dicer cleavages.

## 2. Results

### 2.1. Structural Motifs in miRNA Precursors Influence Dicer Cleavage Precision

Our previous analyses demonstrated that the pre-miRNA structure influences miRNA length diversity, *i.e.*, the formation of miRNAs of different lengths from different precursors [4]. Here, we intended to determine whether structural motifs of pri-miRNAs that occur directly at Drosha and Dicer cleavage sites affect cleavage precision. To address this issue, we used published small RNA deep-sequencing data of the human HEK293T cell line that were obtained with the use of the Illumina platform and provided by David Galas [19]. We selected from these data the most abundant miRNA variants for each miRNA that had a minimum of five sequencing reads to reconstruct the positions of Drosha and Dicer cleavage sites within the predicted structures of pri-miRNA. We created two groups of pri-miRNAs depending on whether a homogeneous or heterogeneous cleavage occurs (Figure S1). We defined homogeneous cleavage as one with a frequency of cleavage at a single position greater than 90%, whereas heterogeneous cleavage is one in which less than 70% of all cuts occur at a single position. This analysis revealed that homogenous Dicer cleavage in the 5' arm of the precursor occurs most often in a secondary structure motif (asymmetrical or symmetrical loop, bulge or terminal loop) whereas heterogeneous Dicer cleavage occurs with almost equal frequency in pre-miRNA hairpin distorted and undistorted at its cleavage site (*p* = 0.0074) (Figure S1). Such a structural bias was not observed for Drosha cleavage (*p* > 0.3). These data suggests that cleavages at one of the four pri-miRNA processing sites may be controlled by local structural factors (Figure S1).

### 2.2. The pri-miRNA Sequence Surrounding the Ends of Mature miRNAs Exhibits Frequency Biases

**Figure 1:**
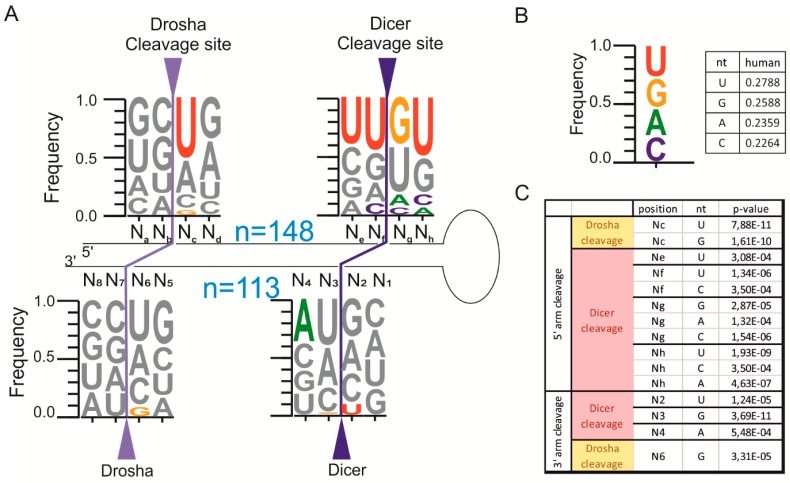
The nucleotide frequencies at hairpin positions adjacent to the Drosha cleavage site (N_a_, N_b_, N_c_, N_d_, N_5_, N_6_, N_7_, and N_8_) and the Dicer cleavage site (N_e_, N_f_, N_g_, N_h_, N_1_, N_2_, N_3_, and N_4_) in the most abundant miRNA variant is presented as a WebLogo sequence. (**A**) For each sequence logo, the y-axis denotes the frequency of that position being a specific nucleotide, with the size of the nucleotide correlating to its frequency. The nucleotides that are significantly different from the background are marked with solid-color letters (*p* < 0.00078, two-sided Fisher’s exact test with Bonferroni correction), and the *p*-values are provided in Figure 1C. The letter n denotes the number of analyzed miRNAs; (**B**) The background was defined as the average frequencies of nucleotides that were identified for the human pri-miRNA sequences that were deposited in miRBase ver. 14 (the excluded miRNAs are described in the Experimental Section). The fraction of each nucleotide is presented on the right; (**C**) *p*-value for the statistically significant differences that were observed between total miRNA population *vs.* background. Positions are designated as in (**A**).

It has been determined in our previous studies [4,20,21,22,23,24] that the patterns of Dicer cleavage are different for different isostructural precursors (*i.e.*, pre-miRNAs having a fully base-paired stem or a stem distorted by similar structural elements) and heterogeneous products of different lengths were observed. These observations focused our attention on the role of the nucleotide sequence in the selection of cleavage sites by Drosha and Dicer. To address this issue, again we took advantage of the same small RNA deep-sequencing data [19]. We used the nucleotide sequences of the most abundant variants of the miRNAs to reconstruct the pri-miRNA sequence in the proximity of the miRNA ends. We analyzed the nucleotide frequencies at two neighboring positions surrounding each miRNA end (positions N_a_, N_b_, N_c_, N_d_, N_e_, N_f_, N_g_, and N_h_ and positions N_1_, N_2_, N_3_, N_4_, N_5_, N_6_, N_7_, and N_8_ in Figure 1A) and compared these frequencies to background nucleotide frequencies (Figure 1B). *p*-values of statistically significant differences between analyzed datasets are shown in Figure 1C.

The results demonstrated that the pri-miRNA nucleotides present at positions corresponding to the first and last nucleotides of the most abundant miRNAs exhibit frequency biases. The distribution of the nucleotides in these positions differs from the background (Figure 1B), and some nucleotides are significantly favored (U residues at positions N_c_ (*p* = 7.88 × 10^−11^) and N_f_ (*p* = 1.34 × 10^−6^), whereas other nucleotides are significantly disfavored (G residues at positions N_c_ (*p* = 1.61 × 10^−10^), N_3_ (*p* = 3.69 × 10^−11^), and N_6_ (*p* = 3.31 × 10^−5^). Our analyses also demonstrate the importance of the nucleotides neighboring the miRNA ends, which may contribute to determining the specificity of Drosha and Dicer cleavage. These features include the following: (1) the reduced frequency of G residues at the 5' end of the miRNA (observed also for nematode and mouse miRNAs [5,18]); (2) the overrepresentation of U residues at the 3' end of the miRNA; (3) the underrepresentation of A and C residues and the enrichment in G and U residues at positions adjacent to the Dicer cleavage site in the 5' arm of the precursor; and (4) the overrepresentation of A residues in the second position of miRNAs derived from the 3' arm of precursors (*p* = 5.48 × 10^−4^).

### 2.3. Homogeneous and Heterogeneous miRNAs Differ in Their End Sequence

**Figure 2:**
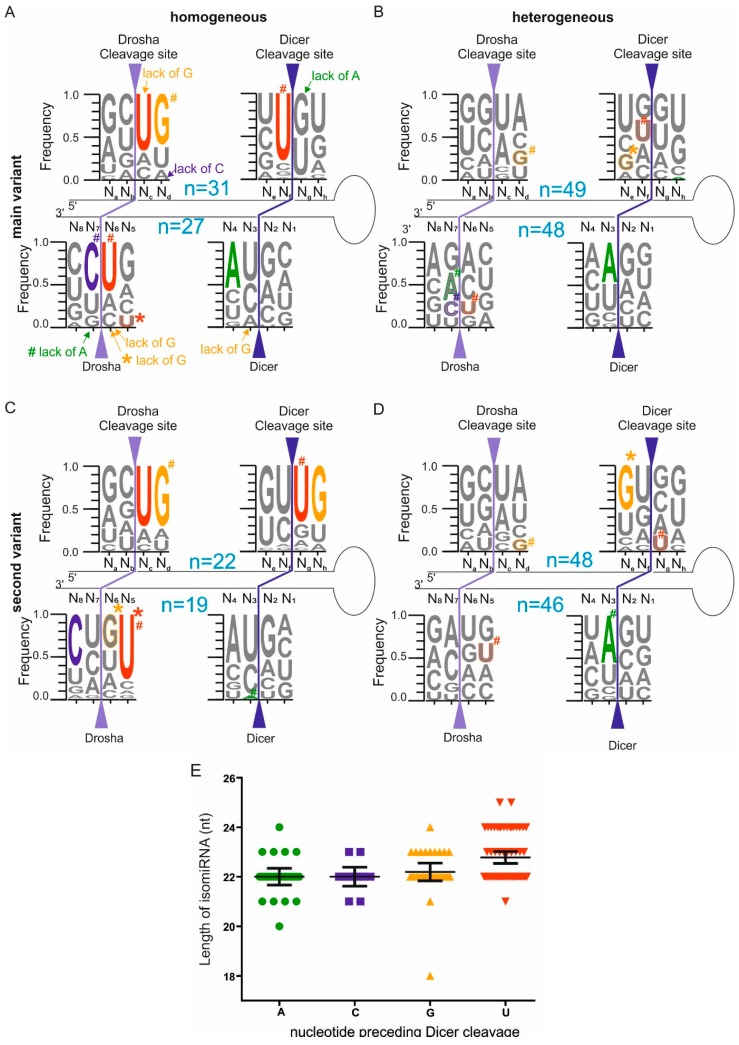
The nucleotide frequencies at hairpin positions surrounding the main (**A**,**B**) and the second-most-frequent (**C**,**D**) miRNA variants among the homogenous (**A**,**C**) and heterogeneous miRNAs (**B**,**D**) are presented as WebLogo sequences. The nucleotides that are significantly different from the background (Figure 1B) are marked with solid-color letters or appropriate designations (*p* < 0.00078, two-sided Fisher’s exact test with the Bonferroni correction). The colored # and outlined letters correspond to nucleotides that are significantly different between the homogeneous and heterogeneous miRNA groups (Part A *vs.* Part B and Part C *vs.* Part D in Figure 2). The colored asterisk (*) and outlined letters correspond to nucleotides that are significantly different between the main- and second-most frequent miRNA variants (Part A *vs.* Part C and Part B *vs.* Part D in Figure 2). All of the statistically significant *p*-values are provided in Table S1. The other designations are the same as in Figure 1; (**E**) The distribution of the miRNA lengths based on the nucleotide specificities of the Dicer cleavages. The column scatter plot and the mean values with a 95% confidence interval are shown. The x- and y-axes denote each isomiR’s terminal nucleotide and its length, respectively. The analyzed isomiRs were derived from the 5' arm of the pre-miRNA whose 5' end was generated through homogeneous Drosha cleavage (regardless of the heterogeneity/homogeneity at the 3' end of the miRNA). Each of the colored symbols represents one isomiR (most frequent).

In the next step, we asked whether characteristic sequence features that exist in the pri-miRNAs may drive Drosha and Dicer to generate homogeneous or heterogeneous miRNAs. To address this question, we again stratified the mature miRNAs into homogeneous and heterogeneous miRNA groups based on the frequency of the main variant (see Experimental Section for details). Then, we analyzed the sequences of the pri-miRNAs from which the homogeneous and heterogeneous miRNAs were derived. Specifically, we determined the frequencies of the nucleotides that surrounded the miRNA ends in the two groups of pri-miRNAs (Figure 2A,B solid-color letters). We have noticed that in both the homogeneous and heterogeneous miRNA groups, certain positions were preferentially occupied by particular nucleotides, whereas the other positions were lacking clear preferences. Specifically, in the pri-miRNA group from which the homogeneous miRNAs were generated (Figure 2A), a lack of G residue was observed at the first position of the miRNA (*p* = 1.27 × 10^−4^). This effect was accompanied by G enrichment at position N_d_ (*p* = 3.44 × 10^−5^), from which C residues were absent (*p* = 7.52 × 10^−4^) (Figure 2A). Greater overrepresentation of U residue in the homogeneous miRNA group compared with the total miRNA population (not divided into groups, Figure 1A) was observed at both ends of the miRNAs derived from the 5' arms of their precursors (positions N_c_ (*p* = 4.61 × 10^−6^) and N_f_ (*p* = 1.56 × 10^−9^)). There was also a lack of A residues at position N_g_ (*p* = 4.11 × 10^−4^) (Figure 2A). The miRNAs derived from the 3' arms of their precursors most frequently ended with U residues (*p* = 7.76 × 10^−4^) and did not contain G residues at their ends (positions N_3_ (*p* = 5.57 × 10^−4^) and N_6_ (*p* = 5.57 × 10^−4^)). Position N_7_ was enriched for C residues (*p* = 4.55 × 10^−5^), whereas position N_4_ was enriched for A residues (*p* = 3.47 × 10^−4^). In the pri-miRNA group from which heterogeneous miRNAs were generated (Figure 2B), enrichment in A residues occurred at position N_3_ (*p* = 2.41 × 10^−4^). This analysis demonstrated that more sequence constraints exist in pri-miRNAs from which homogeneous miRNAs were generated than in pri-miRNAs that gave rise to heterogeneous miRNAs.

The sequence bias that was observed in both homogeneous and heterogeneous miRNAs does not provide straightforward information as to whether these two groups differ significantly with respect to the nucleotides preferred for Drosha and Dicer cleavage. To identify characteristic sequence features that distinguish between homogeneous and heterogeneous groups of miRNAs, we directly compared the nucleotide occupancies at relevant positions in these two groups of pri-miRNAs. The differences that were statistically significant are marked by # in Figure 2. In the group of pri-miRNAs whose 5' arm gives rise to the main homogeneous miRNA variants (Figure 2A), strong enrichment in G residues occurred at position N_d_ (*p* = 2.22 × 10^−4^). By contrast, in pri-miRNAs giving rise to the main heterogeneous miRNA variants, a reduced G occupancy was observed at position N_d_ (Figure 2B). In the pri-miRNAs from which the homogeneous miRNAs were derived (Figure 2A), enrichment in U residues was also observed at position N_f_ (*p* = 2.7 × 10^−6^) (Figure 2A). The 3' terminus of the miRNA (position N_6_) was also preferentially occupied by U residues (*p* = 2.51 × 10^−4^), but position N_7_ was preferentially occupied by C residues (*p* = 7.07 × 10^−4^), and A residues were absent from position N_7_ (*p* = 3.07 × 10^−4^). Together, these features could be considered sequence determinants of a single cleavage site selection by Drosha and Dicer.

Our analyses indicate that more sequence determinants of precise cleavage exist at Drosha sites than at Dicer sites (Figure 2A,C). However, at the Dicer sites, the profound overrepresentation of U residues at position N_f_ (Figure 2A) indicates a strong pressure for miRNAs to terminate with U residues. Therefore, to determine whether the pre-miRNA sequence surrounding the Dicer cleavage site contributes to the site selection, we plotted the miRNA length against its 3' terminal nucleotide (Figure 2E). This analysis was performed for the most frequent miRNA variant that was generated from the 5' arm of the pre-miRNAs that had homogeneous 5' ends. The analysis revealed that the mean length of the main variant that is generated by Dicer does not differ among the products ending with an A, C or G residues (22, 22, and 22.2 nt, respectively; Figure 2E). However, the mean length of the products ending with U residues was higher (22.7 nt), and this difference is statistically significant (*p* = 4 × 10^−4^). This finding may indicate that Dicer, which senses the pre-miRNA substrate to select the optimal cleavage site, tends to cleave after U residues even if the released product is somewhat longer.

### 2.4. Different Abundances of miRNA Variants Reflects the Sequence Preference of Drosha and Dicer Cleavage

It is very rarely observed that only one miRNA variant is generated by Drosha and Dicer cleavage from one pri-miRNA arm. In most cases, the main miRNA variant is accompanied by other isomiRs of lower abundance. In the pri-miRNA group from which the homogeneous miRNAs were generated, the second-most-frequent variant comprised less than 10% of all of the reads mapping to the analyzed miRNA, whereas in the group of pri-miRNAs generating heterogeneous miRNAs, this variant may comprise up to 50% of all of the reads representing each miRNA. Therefore, it is plausible to assume that differences in the abundance of the main miRNA variant and other less-abundant variants may be attributed to the sequence preferences of Drosha and Dicer cleavage. Because small differences in the relative abundance exist between the main- and second-most frequent miRNA variants among the heterogeneous miRNAs and respective large differences occur in the homogeneous miRNAs, we wondered whether there were any characteristic features of the pri-miRNAs sequences preferentially recognized by Drosha and Dicer. To address this issue, we directly compared the nucleotide occupancies at the relevant positions in the analyzed groups of pri-miRNAs, and the differences that were statistically significant are marked by an asterisk (*) in Figure 2 (Table S1). A low frequency of U residues at position N_5_ (*p* = 1.76 × 10^−5^) and a lack of G residues at position N_6_ (*p* = 2.9 × 10^−4^) are characteristic of the main variants of homogeneous miRNAs (Figure 2A). By contrast, the second-most-frequent variants among the homogeneous miRNAs exhibit strong enrichments in U and G at positions N_5_ and N_6_, respectively (Figure 2C). Within the heterogeneous miRNA group, there is only one significant difference: the predominance of G residues at position N_e_ (*p* = 7.46 × 10^−4^) in the second-most-frequent miRNA variant but not in the main variant (Figure 2B,D). These results indicate that the specific sequence features that are characteristic for the second-most-frequent variant among the homogeneous miRNAs may be responsible for its relatively low abundance.

### 2.5. Drosha or Dicer—Which Cleaves More Precisely?

**Figure 3:**
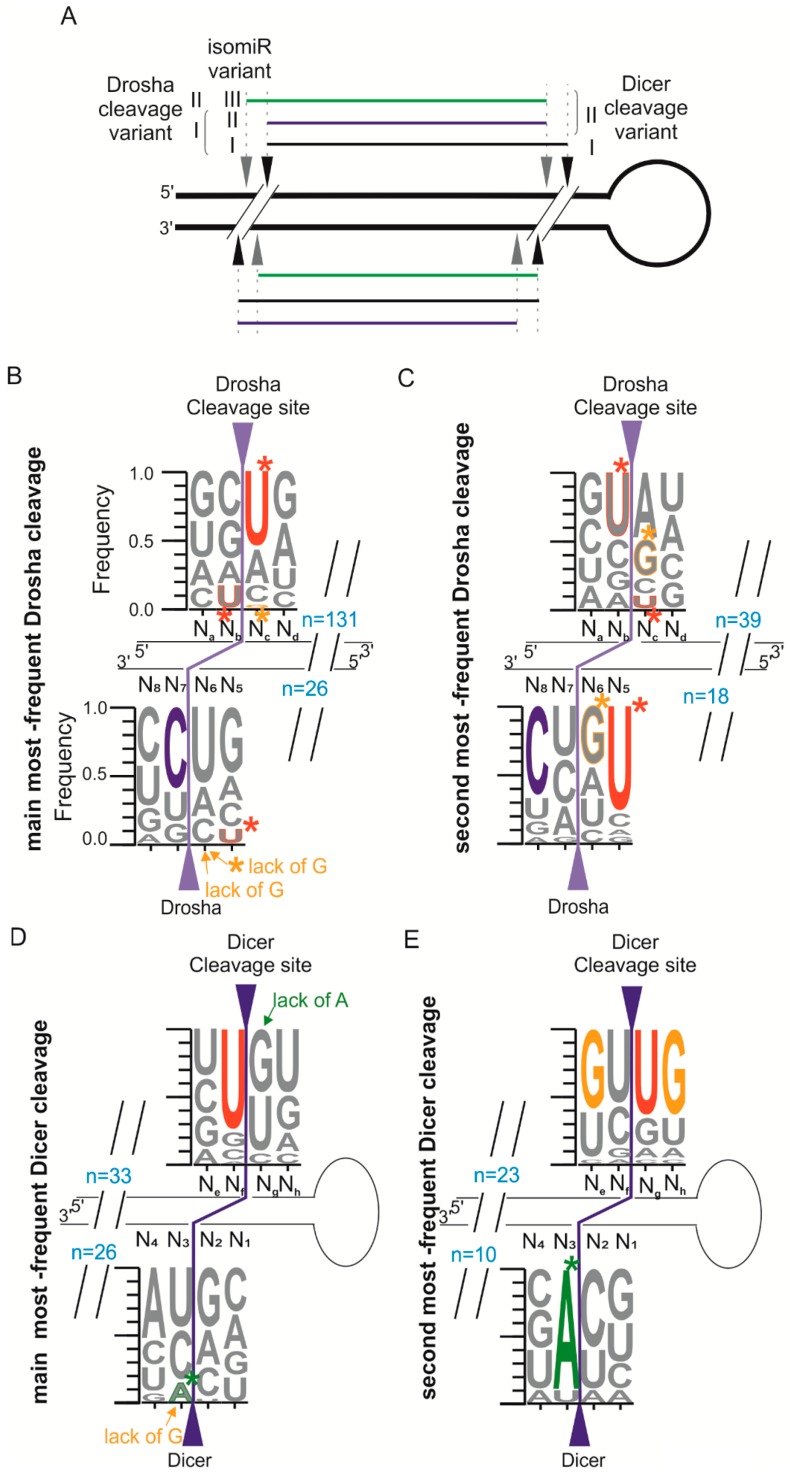
Sequence specificities of the nucleases Drosha and Dicer. (**A**) Relationship between the cleavage sites and the generated miRNA variants. A similar description as for the 5' arm should be applied to the 3' pri-miRNA arm; (**B**–**D**) The nucleotide frequencies that were observed at hairpin positions surrounding the Drosha and Dicer cleavage sites are presented as WebLogo sequences. Separate analyses were performed for the main (**B**) and second-most-frequent (**C**) Drosha cleavage sites within the group of miRNAs with a homogenous end that was generated by Drosha (regardless of the homogeneity or heterogeneity of the end that was generated by Dicer). For miRNAs with an end that was generated by the main homogenous Drosha cleavage (analyzed in **B**), the subsequent Dicer cleavage sites were analyzed both for the main (**D**) and second-most-frequent (**E**) homogeneous Dicer cleavage sites. The other designations are the same as in Figure 2.

The two most-abundant isomiRs that are generated from one pri-miRNA arm may originate either from homogeneous Drosha cleavage and heterogeneous Dicer cleavage or vice versa, or from both Drosha and Dicer heterogeneous cleavage (Figure 3A). What remains to be determined is the relative contributions of imprecise Drosha and Dicer cleavage to isomiR formation.

A comparison of the nucleotide frequency patterns at positions surrounding the termini of the main- (Figure 2A) and second-most (Figure 2C) frequent variants of the homogeneous miRNA that were generated from the 5' arm of pre-miRNA clearly indicates that the patterns surrounding Drosha cleavage sites are very similar and that both variants start with the UG sequence. When we examined the two positions within each pri-miRNA sequence cleaved by Drosha most often (Figure 3B,C), we observed that the patterns are different and that only the main Drosha cleavage produces isomiRs beginning with UG. The second Drosha cleavage produces miRNA starting with AU, which was not represented in the analyses of the main and second miRNA variants presented in Figure 2. This result indicates that the main and second-most-frequent miRNA variants that are generated from the 5' arm of pri-miRNA result from Drosha cleavage at the same site. A similar comparison of the nucleotide frequency pattern for the Dicer cleavage site in the 5' arm of precursor (Figure 2A,C) indicates that these patterns are substantially different. Furthermore, the second-most-frequent miRNA variant, which ends within the sequence 5'-GU↓UG-3' (Figure 2C), is shifted towards 1-nucleotide (nt)-shorter products compared with the main miRNA variant, which ends within a 5'-UU↓GU-3' sequence (Figure 2A). These results demonstrate that the heterogeneity of the isomiRs generated from the 5' arm of the precursors arises primarily from imprecise Dicer cleavage (Figure 3D,E).

A similar comparison performed for the miRNA that is generated from 3' arm of precursor shows that both the main- and second-most frequent variant of homogeneous miRNAs (Figure 2A,C) begin with the UA sequence. When examining the cleavage site from the perspective of the enzyme, more surprising is the fact that the second-most frequent cleavage by Dicer produces isomiRs beginning predominantly with A (Figure 3E), which is not represented in a group of the second variant of homogeneous miRNA that begins least frequently with A residues (Figure 2C). This observation suggests that the two most abundant homogeneous miRNA variants (Figure 2A,C) are generated by Dicer cleavage at the same site (Figure 3D). Similar to the shift in the Dicer cleavage site that occurs at the 3' end of miRNA in the 5' arm of precursor, a shift also occurs at the 3' end of the miRNAs in the 3' arm of the precursor where Drosha perform a cleavage. The main miRNA variant ends with the sequence 5'-GU↓CC-3' (Figure 2A), and the second-most-frequent miRNA variant ends with the sequence 5'-UG↓UC-3' (Figure 2C). Thus, the sequence patterns surrounding the miRNA ends indicate that the second-most frequent miRNA variant is 1-nt shorter than the main miRNA variant, which agrees with the observations of other authors who analyzed the lengths of the miRNA variants [5,6]. These results suggest that the heterogeneity of the isomiRs generated from the 3' arm of the precursors is determined by imprecise Drosha cleavage rather than imprecise Dicer cleavage. 

## 3. Discussion

In our previous studies, we analyzed the sources of miRNA length diversity from the perspective of the miRNA precursor structure as well as the role of proteins that cooperate with Dicer in miRNA biogenesis [4,20,21,22,23,24]. We demonstrated that the patterns of Dicer cleavage were different for different isostructural precursors. The pre-miRNAs with their hairpin stem distorted by similar structural motifs still gave raise to heterogeneous products of different lengths [4]. These observations turned our attention to the role of the nucleotide sequence in the selection of cleavage sites by Drosha and Dicer, an issue that has not been addressed and answered by analyzing human data. Similar studies focusing on sequence features of pri-miRNAs, pre-miRNAs and miRNAs revealed some sequence biases at Drosha and Dicer cleavage sites in nematodes, mice, and flies [5,18,25].

The results of our analysis of the nucleotide frequencies surrounding the sites of Drosha and Dicer cleavage showed a non-uniform distribution of nucleotides not only at the first and last positions of the miRNAs [5,18] but also at the neighboring positions. It is intriguing to speculate that sequence biases at miRNA ends and at nucleotide positions neighboring the miRNA ends (Figure 1) could be considered features contributing to cleavage site selection by Drosha and Dicer, especially when considering the existence of RNA sequence determinants and antideterminants of RNaseIII cleavages in bacteria and yeast [26,27,28,29,30]. The results showing sequence preferences and dispreferences at Drosha and Dicer cleavage sites (Figure 1) corresponded with the results of analogous analyses of mouse miRNAs expressed in two cell types: embryonic stem cells and differentiated cells (Figure S2).

Our study shows that the predominant cleavage sites for Drosha and Dicer rarely contain G residues at the end of a miRNA duplex, and their cleavages retain G residues in the cleaved-off fragments flanking the miRNA duplex. The experimental results reported previously [16] confirm that observation. The results of our analysis also indicate a strong preference for the U residue at both of the miRNA ends. While Ago2 discriminates the 5' end of miRNA [13] we observe sequence bias at different positions within Drosha and Dicer cleavage sites. This suggests that Ago binding, which favors miRNAs with U at their 5' end [13], may not be the only step that generates the U bias. This bias may also derive from the sequence preferences of the cleavage performed by the ribonucleases Drosha and Dicer.

We interpreted the profound overrepresentation of U residues at the last position of miRNA (Figure 3B,D) to be a consequence of RNase III cleavage preferences. However, we cannot rule out the possibility that a small fraction of isomiRs have a U residue at their 3' end not as a result of the sequence preferences of Drosha and Dicer cleavages but rather as a result of post-cleavage modifications that alter this end of the miRNA [31]. It has been reported that both the miRNA and pre-miRNA 3' ends may be extended by various nucleotidyl transferases, resulting in non-templated sequence heterogeneity [32]. A previous analysis of mouse small RNA deep-sequencing data reported that ~16% of all miRNAs reads were extended by 1 nt [33], with U and A additions being most frequent [34,35]. The former occurs in miRNAs that are generated from the 3' arm of the pre-miRNA [7,33], and the latter occurs in miRNAs from the 5' pre-miRNA arm. Our input data, however, were depleted of isomiRs whose sequences did not match their precursors [19], so only those modifications of miRNA and pre-miRNA 3' ends that match a precursor sequence by chance could be misinterpreted as resulting from sequence preferences of Drosha and Dicer. As we observed a U nucleotide highly overrepresented at the miRNA 3' end generated from 5' pre-miRNA arm, which is a nucleotide not preferentially added by nucleotidyl transferases to this site as previously reported [7,33], we regard this effect as having no significant impact on the results of our analyses. Likewise, we considered trimming processes of miRNAs by cellular exonucleases [12] to have minor effects on the outcome of our analysis.

**Figure 4:**
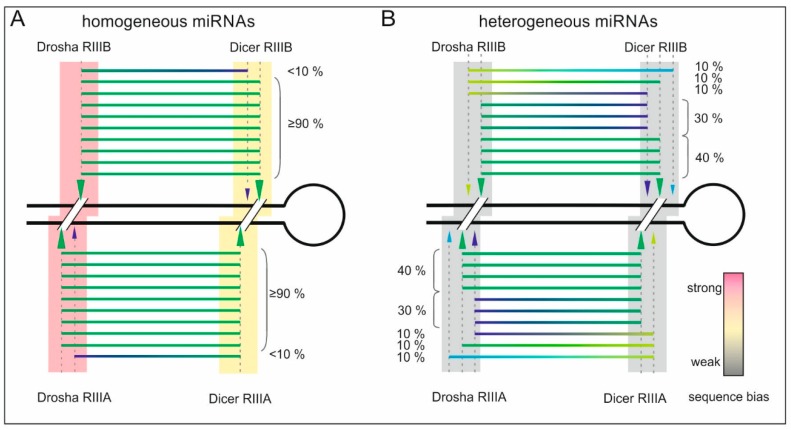
Schematic representation of the pool of isomiRs released from (**A**) homogeneously and (**B**) heterogeneously processed pri-miRNAs. The cleavage sites of the Drosha and Dicer RNase domains (RIIIA and RIIIB) are indicated with arrowheads whose size corresponds to the intensity of cleavage at these positions. The background colors at different cleavage sites indicate the occurrence of sequence bias at these sites, which affects the generation of homogeneous and heterogeneous miRNAs (according to the legend).

It is also apparent from our study that Drosha cleavage at a single site is more sequence-dependent than cleavage by Dicer. More sequence constraints exist in pri-miRNAs from which homogeneous miRNAs are generated, and these constraints are likely to influence the precision of Drosha and Dicer cleavage (Figure 4). We propose that the cleavage products generated by the RIIIA and RIIIB domains of Drosha and Dicer differ in their degree of precision. The RIIIA domain of Drosha cleaves at more scattered positions, whereas the RIIIA domain of Dicer makes more specific cuts. Conversely, the RIIIB domain of Drosha makes more specific cleavages, whereas the RIIIB domain of Dicer performs less specific cuts. Such differences in the degree of cleavage precision shown by these domains may be explained by the different sequence and structural features that are recognized by the RIIIA and RIIIB domains at the Drosha and Dicer cleavage sites (Figure 2 and Figure 3).

The present study demonstrates that the presence of local structural motifs of miRNA precursors has also an impact on the precision of Dicer cleavage. The fact that a homogeneous Dicer cleavage in the 5' arm of a pre-miRNA occurs most often within a stem distorted by secondary structure motifs suggests that flexibility of RNA may allow precise positioning of nucleotides to be cleaved by Dicer.

In addition to extending our knowledge of the sequence/structure preferences of Drosha and Dicer cleavage [5,18], this study also sheds light on the issue of homogeneous and heterogeneous miRNAs formation by Drosha and Dicer (Figure 4). The occurrence of homogeneous miRNAs is very rare. By contrast, the formation of heterogeneous miRNAs having higher regulatory potential is more desirable from the perspective of “cell economy”, less restricted, and indeed more common.

## 4. Experimental Section 

### 4.1. Small RNA Dataset

Deep-sequencing data for the human HEK293T cell line that were obtained with the use of the Illumina platform were provided by Dr. David Galas. Detailed information on the sample preparation and NGS data processing is described in Lee *et al.* [19]. It should be noted that we excluded 19 human miRNAs (Table S3) from the dataset that were negatively validated in the miRBase (ver. 20) [36]. In addition, based on previously published studies [37,38], we deleted isomiRs from the dataset that were mirtrons that bypass Drosha processing, as well as miR-451, which is produced by a Dicer-independent pathway [39] (Table S4). To minimize the effect of sequencing errors on the final result, isomiRs that were represented by fewer than five sequencing reads were excluded. Eliminating miRNAs having low read count by setting a threshold is a common practice when analyzing deep-sequencing data (compare with [5,40,41]). Additional abundance criteria were as follows: (I) if there were 3 or more isomiR, then the sum of their reads had to be be greater than 14, and the number of reads for most abundant miRNA must be greater than 5; (II) if there were 2 isomiRs then the sum of their reads had to be greater than 14; (III) if there were only one miRNA variant, it must have more than 14 reads. This approach gave us groups of homogeneous and heterogeneous miRNAs that allowed us to draw statistically significant conclusions. From 436 miRNA being expressed in HEK293, after excluding miRNA listed in Supplementary Tables S3 and S4, and after applying the abundance threshold described above, a set of 261 human miRNAs was subjected to our analyses. Each main isomiR for the different miRNAs was weighted equally, regardless of the number of sequencing reads.

For the analyses of mouse miRNA, we used deep-sequencing of embryonic stem cells and differentiated cells data provided by David Galas [5]. As for human miRNAs, from mouse dataset we excluded 22 miRNAs negatively validated in the miRBase (ver. 20) (Table S3) and deleted 7 miRNAs that are generated in Drosha or Dicer independent process (Table S4). Altogether, from 465 mice miRNAs being expressed in embryonic stem cells, 296 miRNAs satisfied our criteria and were subjected to further analyses. In mouse differentiated miRNA dataset, there were 593 miRNA expressed, while 415 miRNAs passed our criteria. 

### 4.2. Structural Analyses

In the structural analysis, we used only miRNAs that were represented in both of the arms of the miRNA precursors to reliably reconstruct the Drosha and Dicer cleavage sites. To obtain the secondary structures of the miRNA precursors, we used the UNAFold software, ver. 3.8 with the default parameters for temperature (37 °C) and sequence type (linear) [42]. As an input, we used the stem-loop sequences of miRNA precursors that were obtained from miRBase (ver. 20) [36]. The folding of a stem-loop with the lowest minimum free energy was always selected for further analysis. Within a sequence of each pri-miRNA, we assigned the sequence of main miRNA variant, followed by the analysis of the secondary structure motif at Drosha and Dicer cleavage sites within a group of homogeneous and heterogeneous miRNAs.

### 4.3. Analysis of the Sequence Composition Surrounding the miRNA Processing Sites

The nucleotide frequencies were depicted in the form of sequence logos using the WebLogo application, version 3.3 [43], with the output providing “probability” on the y-axis (*i.e.*, nucleotide frequency). The nucleotide frequencies at the positions on either side of the presumed processing site (4-nt window—2 positions each side of a cleavage site ) (N_a_, N_b_, N_c_, N_d_, N_5_, N_6_, N_7_, and N_8_—positions surrounding Drosha cleavage sites; N_e_, N_f_, N_g_, N_h_, N_1_, N_2_, N_3_, and N_4_—positions surrounding Dicer cleavage sites) were determined for the main- and second-most frequent miRNA variants. A two-sided Fisher’s exact test with Bonferroni correction (R software package) was used to identify the nucleotide frequencies at the queried positions that differed from the background in a statistically significant manner. The background (Figure 1B) was calculated using the human miRNA precursor sequences (ver. 14.0) from miRBase [44] after excluding the miRNAs that are described in the section “Small RNA dataset”; the same version of miRBase was used in an NGS sequence alignment [19]. The differences between the analyzed dataset and the background were considered statistically significant for *p* < 0.00078 (0.05/64 after Bonferroni correction). We have also done preliminary analyses with a use of 8-nt window (4 nt positions at each side of a cleavage site), instead of 4-nt window (2 nt positions at each side of a cleavage site) but only a few nucleotides have been statistically significant changed compared to the background (data not shown). Since the majority of statistically enriched or reduced nucleotides lied within the 4-nt window, we used this parameter for the rest of our analyses. When indicated, the nucleotide frequencies of the homogenous miRNA positions were compared with their respective positions on the heterogeneous miRNAs, and the statistically significant differences (two-sided Fisher’s exact test with Bonferroni correction) are indicated in Figure 2 with a hash symbol (^#^). When indicated, the nucleotide frequencies of the main miRNA variants were compared with their respective positions on the second-most frequent miRNA variants, and the statistically significant differences (two-sided Fisher’s exact test with Bonferroni correction) are indicated in Figure 2 and Figure 3 with an asterisk (*). In both of the cases, the *p*-value thresholds were applied in the same way as in the comparison between the analyzed datasets and the background. 

### 4.4. Sequence Features of Homogeneous and Heterogeneous miRNAs

To analyze the sequence features of the homogeneous and heterogeneous miRNAs, we divided all of the isomiRs into two groups based on the frequency of the most frequent isomiR. The homogeneous miRNAs were the main miRNA variants that were more than 90% abundant among all of the miRNA variant reads that were detected for each miRNA that had at least 5 reads (Figure 4A). The heterogeneous miRNAs were the main miRNA variants that were less than 70% abundant among all of the miRNA variant reads that were detected for each miRNA that had at least 5 reads (Figure 4B). The group of isomiRs that had 70%–90% abundance of the main miRNA among all of the miRNA variants was not analyzed because of the high degree of divergence in this group. Using the above ranges, the second-most-frequent variant among the homogeneous miRNAs was less than 10% abundant among all of the miRNA variant reads that were detected for each miRNA (Figure 4A). Note that a second homogeneous miRNA variant was observed only for those miRNAs for which the main isomiR was not a single variant (100% abundance). Among the heterogeneous miRNAs, the second-most-frequent isomiR may have been nearly as abundant as the main variant.

### 4.5. Analyses of Drosha and Dicer Cleavage Specificities

To analyze the sequence features surrounding the Drosha and Dicer cleavage sites, we pooled populations of isomiRs with ends that were generated by the same Drosha cleavage. We performed this analysis on the groups of isomiRs that were derived from the main (frequency of cleavage greater than 90%) and second-most-frequent (frequency of cleavage less than 10%) Drosha and Dicer cleavages. The Drosha cleavage site was analyzed regardless of the Dicer cleavage site, whereas the Dicer cleavage site was analyzed on the homogeneous products of Drosha cleavage. The frequencies of the main- and second-most-frequent cleavages were calculated from the pool of isomiRs mapping to the analyzed miRNAs that began or ended at the same position (Figure 3A).

### 4.6. Analyses of the Dicer Product Lengths

The length of the Dicer cleavage products was determined for the main isomiR variant (*n* = 131) that was derived from the 5' arm of the precursor whose 5' end was generated by homogeneous Drosha cleavage. For each isomiR, the length of the miRNA and the nucleotide of the last position of the miRNA were analyzed. The differences between the average lengths of products ending with A, C, G or U were calculated using an analysis of variance (ANOVA) (GraphPrism, Software, Inc., San Diego, CA, USA).

### 4.7. Data Mining and Statistics

Analyses of the isomiRs and miRNA precursor sequences and structures were performed using a set of in-house scripts that were written in Python and Perl. Statistical analyses of the nucleotide frequencies were conducted using in-house scripts that were written in the R programming language using the built-in statistical function for the Fisher’s exact test. All of the other statistical analyses were performed using GraphPad (GraphPad Prism, Software, Inc.), and the statistically significant *p*-values are provided in Figure 1 and in Tables S1 and S2.

## Supplementary Materials

Supplementary materials can be found at http://www.mdpi.com/1422-0067/16/04/8110/s1.
