# Supplementary material for: Sequence Features of Drosha and Dicer Cleavage Sites Affect the Complexity of IsomiRs
Source: Int J Mol Sci. 2015 Apr 10;16(4):8110–27. doi: 10.3390/ijms16048110 (PMC4425070; doi:10.3390/ijms16048110)
Supplement: Supplementary file 1 [file ijms-16-08110-s001.zip › ijms-78242-Supplementary Information.pdf]

# Supplementary Information

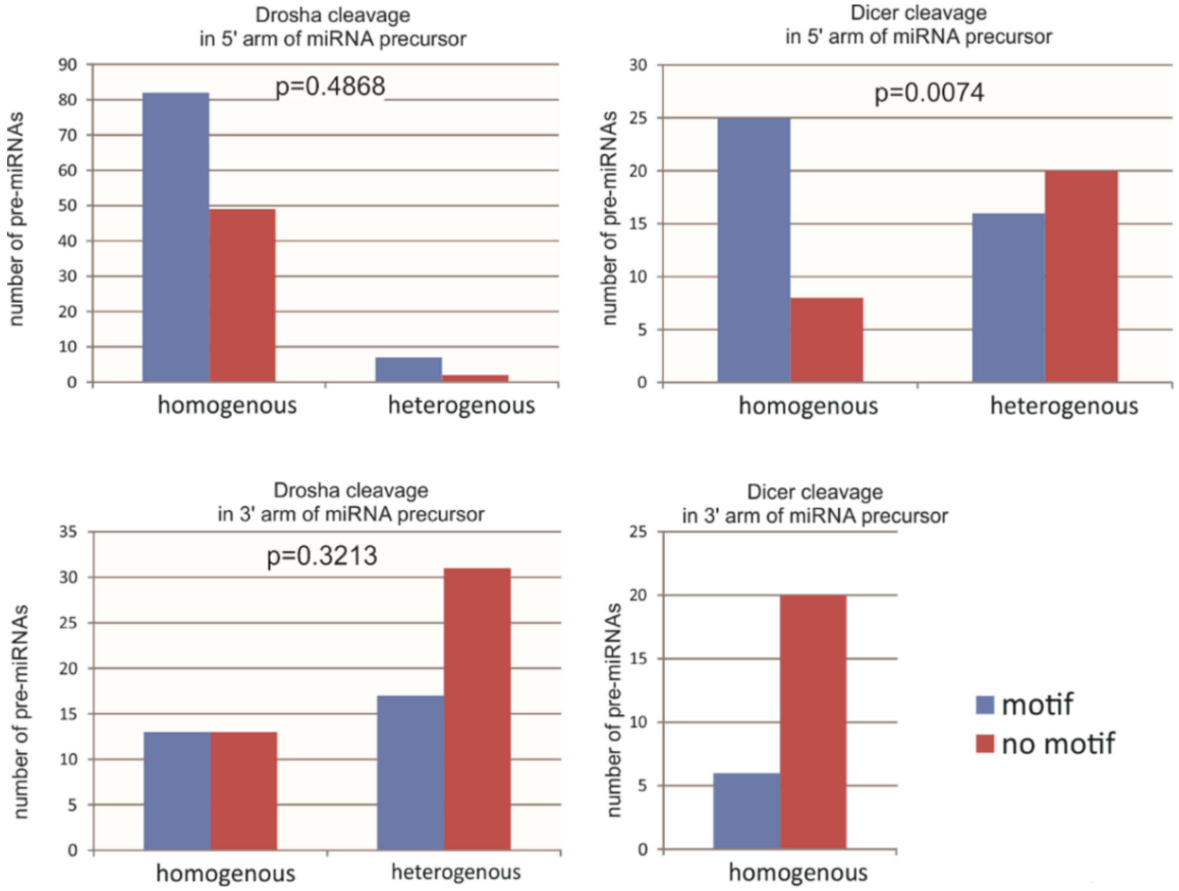


**Figure S1.** Structural characteristics of the homogeneous and heterogeneous Drosha and Dicer cleavage sites. The difference in the number of precursors that were cleaved homogeneously and heterogeneously by Drosha and Dicer in the pri-miRNAs that had their cleavage site disrupted by structural motifs or that had no structural motifs at this site is shown. The statistical significance of this difference was verified by a two-sided Fisher's exact test. Note that when homogeneous Drosha cleavage occurred in the 3' arm of precursor, homogeneous Dicer cleavage also occurred at this hairpin arm; therefore, no heterogeneous cleavages were analyzed.

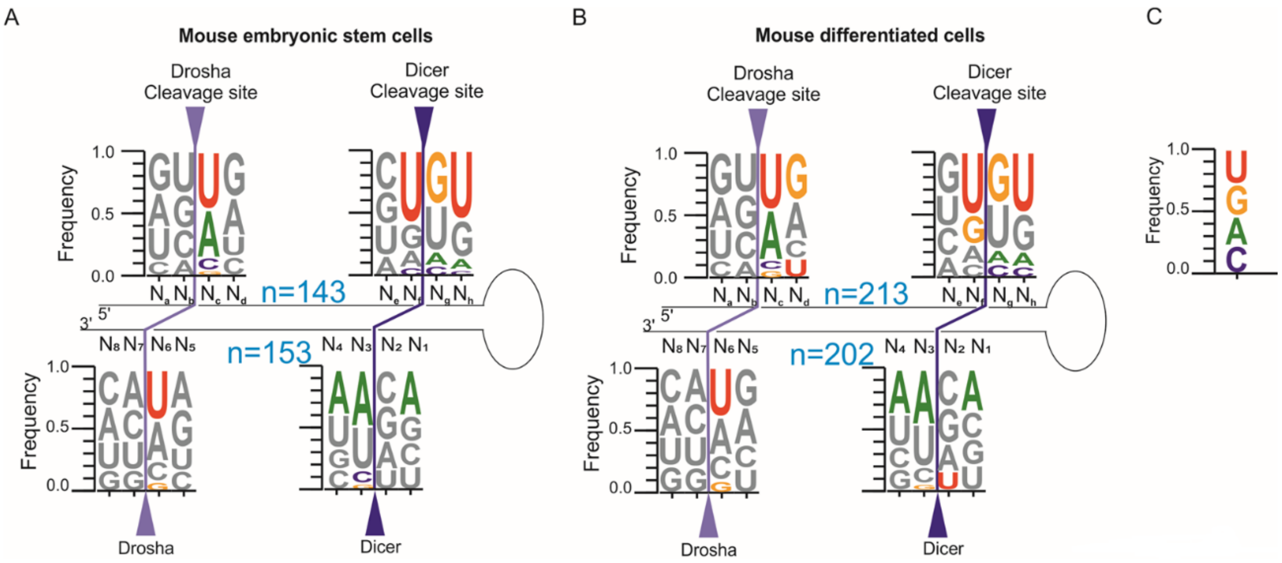


**Figure S2.** The nucleotide frequencies at hairpin positions adjacent to the Drosha cleavage site ($N_a$, $N_b$, $N_c$, $N_d$, $N_5$, $N_6$, $N_7$, and $N_8$) and the Dicer cleavage site ($N_e$, $N_f$, $N_g$, $N_h$, $N_1$, $N_2$, $N_3$, and $N_4$) in the most abundant miRNA variant is presented as a WebLogo sequence for mouse embryonic stem cells (**A**) or mouse differentiated cells (**B**). For each sequence logo, the *Y*-axis denotes the frequency of that position being a specific nucleotide, with the size of the nucleotide correlating to its frequency. The nucleotides that are significantly different from the background (**C**) are marked with solid-color letters ($p < 0.00078$, two-sided Fisher's exact test with Bonferroni correction). The letter n denotes the number of analyzed miRNAs.

**Table S3.** List of human miRNAs that were excluded from the NGS dataset based on the miRBase ver. 20 validation.

| Name | Comments (from miRBase) |
|---|---|
| **Human** | |
| hsa-miR-220a<br>hsa-miR-220b<br>hsa-miR-220c | miR-220 was predicted computationally by the Bartel group, and expression demonstrated in zebrafish (Lim *et al.* [45]). Expression of miR-220 has never been demonstrated in any other animal, but annotation was transferred to other vertebrates by similarity. Subsequent work strongly suggests that miR-220 is not a miRNA, and it is therefore withdrawn by the authors and all miR-220 sequences are deleted from miRBase. |
| hsa-miR-453 | miR-453 is processed from the 5p arm of miR-323b. The entries are merged. |
| hsa-miR-511-2 | Two miR-511 sequences in miRBase 19 map to only one locus in the patched GRCh37.p10 genome assembly. The entries are merged in miRBase 20. |
| hsa-miR-886 | The annotated mature sequence is a fragment of Vault RNA. |
| hsa-miR-1201 | The miRNA overlaps an annotated snoRNA (SNORD126, Rfam 10.0, Infernal 1.0). The patterns of sequence conservation support the snoRNA annotation, so this sequence is likely not a miRNA. |
| hsa-miR-1259 | The miRNA overlaps an annotated snoRNA (SNORD12, Rfam 10.0, Infernal 1.0). The patterns of sequence conservation support the snoRNA annotation, so this sequence is likely not a miRNA. |
| hsa-miR-1274a | The putative mature miR-1274 sequence is a fragment of a Lys tRNA. |
| hsa-miR-1274b | The putative mature miR-1274 sequence is a fragment of a Lys tRNA. |
| hsa-miR-1280 | Schopman *et al.* [46] show that the sequence annotated as miR-1280 is likely to be a fragment of a tRNA, and so is removed from the database. |
| hsa-miR-1308 | The mature miR-1308 is a fragment of a tRNA, and is therefore removed. |
| hsa-miR-720 | Schopman *et al.* [46] show that the sequence annotated as miR-720 is likely to be a fragment of a tRNA, and so is removed from the database. |
| hsa-miR-1826 | The annotated mature sequence is a fragment of 5.8S rRNA. |
| hsa-miR-1974 | miR-1974 maps to the Mt genome, and overlaps a Mt tRNA sequence so is removed. |
| hsa-miR-1975 | The annotated mature sequence is a fragment of Y RNA. |
| hsa-miR-1977 | miR-1977 maps to the Mt genome, and overlaps a Mt tRNA sequence so is removed. |
| hsa-miR-1978 | miR-1978 maps to the Mt genome, and overlaps a Mt tRNA sequence so is removed. |
| hsa-miR-1979 | The mature miR-1979 is a fragment of a Y RNA. |
| **Mouses** | |
| mmu-miR-464 | The pattern of deep sequencing reads from the putative miR-464 locus is not consistent with miRNA processing—many reads overlap the loop region, and are tiled across the whole hairpin. |
| mmu-miR-685 | miR-685 is found to be a fragment of RNase P RNA, so is removed from the database. |
| mmu-miR-685 | miR-685 is found to be a fragment of RNase P RNA, so is removed from the database. |

**Table S3.** *Cont.*

| Name | Comments (from miRBase) |
|---|---|
| **Mouse** | |
| mmu-miR-689-1 | The annotated miRNA sequence is a fragment of rRNA. |
| mmu-miR-689-2 | The annotated miRNA sequence is a fragment of rRNA. |
| mmu-miR-720 | Schopman *et al.* [46] show that the sequence annotated as miR-720 is likely to be a fragment of a tRNA, and so is removed from the database. |
| mmu-miR-715 | The pattern of deep sequencing reads from the putative miR-715 locus is not consistent with miRNA processing -- many reads overlap the loop region, and are tiled across the whole hairpin. Calabrese *et al.* [47] also challenge this annotation, suggesting that it derives from the pre-rRNA transcript. |
| mmu-miR-805 | miR-805 maps to the Mt genome, and overlaps a Mt tRNA sequence so is removed. |
| mmu-miR-220 | miR-220 was predicted computationally by the Bartel group, and expression demonstrated in zebrafish. Expression of miR-220 has never been demonstrated in any other animal, but annotation was transferred to other vertebrates by similarity. Subsequent work strongly suggests that miR-220 is not a miRNA, and it is therefore withdrawn by the authors and all miR-220 sequences are deleted from miRBase. |
| mmu-miR-297a-5 | miR-297a maps many times to the mouse genome. Sequences without support for mature miRNAs from both arms by deep sequencing are deleted. |
| mmu-miR-297a-6 | miR-297a maps many times to the mouse genome. Sequences without support for mature miRNAs from both arms by deep sequencing are deleted. |
| mmu-miR-1186a<br>mmu-miR-1196<br>mmu-miR-1935<br>mmu-miR-1965 | The sequences annotated as miR-1965, miR-1935, miR-1186 and miR-1196 have been shown to derive from DGCR8-independent processing of a long hairpin formed by a transcribed Alu/B1 SINE element. These sequences are therefore likely to be mis-annotations. |
| mmu-miR-1937a | The sequence annotated as mature miR-1937 is a fragment of tRNA, and is therefore not a likely to be a bona fide miRNA. |
| mmu-miR-1939 | The sequence annotated as mature miR-1939 is a fragment of mouse Gly-tRNA, and is therefore not a likely to be a bona fide miRNA. |
| mmu-miR-1944 | The pattern of deep sequencing reads from the putative miR-1944 locus is not consistent with miRNA processing—many reads overlap the loop region, and are tiled across the whole hairpin. |
| mmu-miR-1937b-1 | The sequence annotated as mature miR-1937 is a fragment of tRNA, and is therefore not a likely to be a bona fide miRNA. |
| mmu-miR-1937c | The sequence annotated as mature miR-1937 is a fragment of tRNA, and is therefore not a likely to be a bona fide miRNA. |
| mmu-miR-1959 | The sequence annotated as mature miR-1959 is a fragment of tRNA, and is therefore not a likely to be a bona fide miRNA. |
| mmu-miR-1274a | The putative mature miR-1274 sequence is a fragment of a Lys tRNA. |

**Table S4.** List of the isomiRs that were mirtrons that were excluded from the NGS dataset

| Name | Comments | References |
|---|---|---|
| | **Human** | |
| hsa-miR-451 | Processing of pre-miRNA is Dicer-independent | [39] |
| hsa-miR-1226 | A mirtron, processing Drosha-independent | [38] |
| hsa-miR-1228 | A mirtron, processing Drosha-independent | |
| hsa-miR-1231 | A mirtron, processing Drosha-independent | |
| hsa-miR-1234 | A mirtron, processing Drosha-independent | |
| hsa-miR-1292 | A mirtron, processing Drosha-independent | |
| hsa-miR-1909 | A mirtron, processing Drosha-independent | |
| hsa-miR-937 | A mirtron, processing Drosha-independent | |
| hsa-miR-939 | A mirtron, processing Drosha-independent | |
| hsa-miR-877 | A mirtron, processing Drosha-independent | [37] |
| hsa-miR-1224 | A mirtron, processing Drosha-independent | |
| hsa-miR-1225 | A mirtron, processing Drosha-independent | |
| hsa-miR-1227 | A mirtron, processing Drosha-independent | |
| hsa-miR-1229 | A mirtron, processing Drosha-independent | |
| hsa-miR-1233 | A mirtron, processing Drosha-independent | |
| hsa-miR-1236 | A mirtron, processing Drosha-independent | |
| hsa-miR-1237 | A mirtron, processing Drosha-independent | |
| hsa-miR-1238 | A mirtron, processing Drosha-independent | |
| | **Mouse** | |
| mmu-miR-451 | Processing of pre-miRNA is Dicer-independent | [48] |
| mmu-miR-1981 | A mirtron, processing Drosha-independent | [38] |
| mmu-miR-1982 | A mirtron, processing Drosha-independent | |
| mmu-miR-668 | A mirtron, processing Drosha-independent | |
| mmu-miR-702 | A mirtron, processing Drosha-independent | |
| mmu-miR-877 | A mirtron, processing Drosha-independent | [37] |
| mmu-miR-1224 | A mirtron, processing Drosha-independent | |
